# Evaluation of Postural Asymmetry and Gross Joint Mobility in Elite Female Volleyball Athletes

**DOI:** 10.2478/v10078-011-0034-9

**Published:** 2011-10-04

**Authors:** Renata Vařeková, Ivan Vařeka, Miroslav Janura, Zdenek Svoboda, Milan Elfmark

**Affiliations:** 1 Department of Natural Sciences in Kinanthropology, Faculty of Physical Culture, Palacky University in Olomouc, Czech Republic; 2 Depatment of Physiotherapy, Faculty of Physical Culture, Palacky University in Olomouc, Czech Republic

**Keywords:** posture, functional scoliosis, muscle imbalance

## Abstract

The purpose of the study was to evaluate marked postural asymmetry and gross joint mobility in elite female volleyball athletes.

Sixty-two Czech and Slovak elite female volleyball athletes (age 20.7±2.03 years, body mass 71.1±6.18 kg, body height 1.804±.0618 m, BMI 21.8±1.78) were examined by an experienced rehabilitation physician. The set of tests included the frontal posture gross examination, the forward bending test from the standing position and the deep squat test. The spiking hand and the presence of any lower extremity injury were estimated by interview. The proportion test, Mann-Whitney test and t-test were used to evaluate statistical significance (p<0.05).

Fifty subjects (80.6%) exhibited “typical” frontal plane posture in which the acromion, scapula and the iliac crest were in a higher position on the left side than on the right, significantly more frequently than all the other patterns (proportion test, p<0.0001). Ninety-eight percent of the subjects with the “LLL pattern” preferred the right arm for spiking (proportion test, p<0.0001). Forty-one subjects (66%) exhibited hypermobility in the forward bending test, significantly more frequently than twenty-one subjects (34%) with normal results (proportion test, p=0.0003). Thirty-four subjects (55%) did not succeed in the deep squat test and hypermobility in the forward bending test paradoxically prevailed in them significantly (proportion test, p=0.004). Restriction in the deep squat test was not linked to obesity, age (t-test, p=0.081) nor knee (proportion test, p=0.85) and ankle injury (Mann-Whitney test, p=0.36) in the past. Significant prevalence of hypermobility in the forward bending test was not surprising because of general body composition and the performance of regular stretching exercises in elite female volleyball athletes. On the other hand, surprisingly, more than half of the subjects did not succeed in the deep squat test. The cause of poor results in the deep squat test could be due to the tightness of the soleus muscle suffering from chronic overloading and/or an inappropriate stretching methods. An inappropriate and/or insufficient compensatory exercise and stretching method or system could be the cause of their marked postural asymmetry as well.

A detailed examination of posture and muscle imbalance performed by an experienced physician or physiotherapist as well as individually tailored compensatory exercises and a stretching system can be strongly recommended to all elite athletes, not only to volleyball players.

## Introduction

Postural asymmetry and disorders of gross joint mobility are common in athletes as well as in the general population.

Posture, defined by Kendall et al. (1993) as “a composite of the positions of all the joints of the body at any given moment”, is very important in and of itself. Vareka and Dvorak (2001) have pointed out the role of optimal posture as the necessary condition for the optimal performance of targeted motion. On the other hand, badposture modifies movement in a non-optimal way. That results in the chaining of disorders in the movement system, originally functionally, structurally later. Shumway-Cook and Woollacott (2000a,b, 2001) defined another important construct, postural control, as the control of the body’s position in space for the purposes of balance and orientation. Posture in adults is a result of previous development and prevailing physical activity. Clinical experience evidences the fact that an appropriate sport can help to build and support good posture, however intensive overloading, moreover asymmetrical, causes many complications including some undesirable postural changes. Random repetition of specific movements in the game and practice can lead to the accumulation of one-sided (over)load, which results in faulty posture. Problematic daily habits of players can worsen this situation (Lewit, 1999). The typical posture of various athletes has been known for a long time. Postural asymmetry in volleyball athletes and others performing overhead movements has been described by many authors, e.g. Krüger-Franke et al. (1996), Henne (1998), Yoo et al. (2001), Burkhart et al. (2003), Wang et al (2004), Voralek et al. (2007), Oyama et al. (2008), Kenanidis et al. (2008) and Kugler et al. (2009).

The testing of gross joint mobility is not only an introduction to the detailed examination of the musculoskeletal system, but gives us a lot of important information for further analysis. Its advantage is not only in saving time in everyday practice, but also in relatively easy and sufficient scaling in research. The main functional disorders of joint mobility are joint hypermobility and joint hypomobility (restriction) The source of these disorders can be found in the joints as well as in the muscles and/or in the control system (Lewit, 1999). The likeliest source of joint mobility disorder in uninjured volleyball athletes is muscle imbalance, defined by Janda (1983) as functional muscle shortness or weakness. The relatively high incidence of muscle imbalance in junior volleyball athletes has been confirmed by Voralek et al. (2007).

At the present time, modern sophisticated devices, including 3D kinematic analyser, isokinetic dynamometry, force platform, electromyography, etc. are used in movement diagnostics (e.g. Wang & Cochrane, 2001; Markou & Vagenas, 2006). However, the classical physical examinations, visual as well as manual, have not lost their importance (Kendall et al., 1993; Lewit, 1999). The main object of the present study is to assess the typical posture asymmetry and imbalance in gross joint mobility in Czech and Slovak elite female volleyball athletes. The collateral target is to demonstrate the potential of some simple test for providing basic and incomplete, but important information about the state of the locomotor system.

## Methods

Informed consent was obtained from all teste d subjects. The examined protocol included a questionnaire and a physical examination.

The study group consisted of sixty-two Czech and Slovak elite female volleyball athletes (age 20.7±2.03 years, body mass 71.1±6.18 kg, body height 1.804±.0618 m, BMI 21.8±1.78) who played for the Czech and/or Slovak volleyball extraleague. The athletes practiced intensively for approximately 10 years - two or three training units per week at the start of their sports career, then one or two per day in more recent years. Verbal question was used to identify the preferred hand for spiking as well as any history of knee surgery and/or ankle sprain.

Physical examination was performed by an experienced rehabilitation physician. Each set consisted of a frontal plane posture examination, a forward bending test (standing) and a deep squat test. Each barefoot subject was dressed in a standard female volleyball player’s uniform including a pair of close-fitting shorts and a T-shirt, standing upright with her feet pelvis-width apart. The examiner was sitting behind the subject and examined her frontal plane posture palpating the (a)symmetrical height of the acromion process, the angles of the inferior scapula angles and the iliac crest, as well as the paravertebral muscles. The Adams test of slight forward bending was performed to evaluate the trunk asymmetry accentuation or redress.

The starting position for the forward bending test was that of upright standing with the feet together and straight knees. The subject bent forward to reach the floor. The results were ranked as: (a) “fingers to navicular bone”, (b) “fingers to floor”, (c) “metacarpal heads to the floor”, and (d)”palms to the floor”. Results (a) and (b) were taken as a general norm (normal and near-normal, respectively). The results (c) and (d) were taken as indicating hypermobility (Lewit, 1999).

The starting position for the deep squat test was an upright standing position with the feet apart at shoulder-width. The subject did a maximum deep squat without lifting the soles of her feet from the floor. Full contact between the hamstring and the gastrocnemius muscle was taken as a norm. An inability to reach the full contact position and/or falling backward was rated as a restriction in the case of the deep squat test.

### Data Management

Data were analyzed using Statistica version 8 (StatSoft CR, Inc.). All the analysed data did not demonstrate a normal distribution with the exception of age. Relative frequency was calculated and the proportional test was used for the most prevalent postural pattern and the others, for the preferred spiking arm, for normal results and hypermobility in the forward bending test, for optimal results or restriction in the deep squat test and for subjects with and without knee surgery. Means and standard deviations were calculated for age and ankle sprain incidence in the past. The t-test was applied in order to estimate the influence of the individual’s age on the deep squat test results. The Mann-Whitney U test was used to compare ankle sprain incidence in subjects who succeeded or failed at the deep squat test. Statistical significance were tested at the alpha=0.05 probability level.

## Results

### Postural Asymmetry and the Spiking arm

We observed the “typical” frontal plane posture with the acromion process, the scapula and the iliac crest higher on the left side than on the right one (Table 1). The prevailing “LLL pattern” was observed in fifty subjects (80.6%), whereas other patterns were observed in twelve subjects only (19.4%)(proportion test, p<0.0001). Ninety-eight percent of the subjects with the “LLL pattern” preferred the right arm for spiking (proportion test, p<0.0001). One subject preferred the right hand for spiking but the left one for writing.

### Gross Joint Mobility

Twenty-one subjects (33.9%) reached the general norm in the forward bending test (Table 2). Forty-one subjects (66.1%) had signs of hypermobility. The difference was significant (proportion test, p=0.0003).

**Figure 1:**
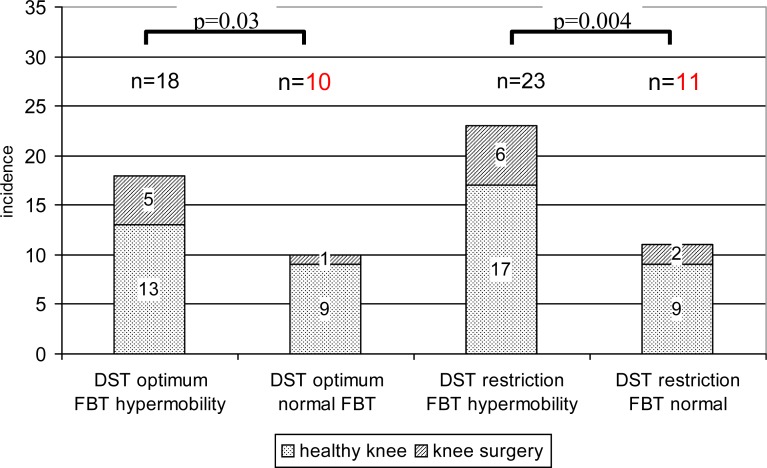
Comparison of the mobility in the forward bending test (FBT) and deep squat test (DST)

Thirty-four subjects (55%) did not succeed at the deep squat test. Hypermobility in the forward bending test paradoxically prevailed (proportion test, p=0.004) (Table 2). Only eleven of them (32.4%) did not demonstrate hypermobility in the forward bending test (Figure 1). To exclude some of the reasons for the deep squat test restriction, we tested the effects of age as well as lower limb injury in the past. The effect of obesity could be excluded because of the presence of a BMI of 21.8±1.78.

The subjects who failed at the deep squat test did not differ in age from the subjects who succeeded at the test (mean=21.0±1.8 or 20.3±2.2 years, respectively; t-test, p=0.081).

There was not a higher incidence of any restriction at the deep squat test in the subjects with a history of knee surgery than in the subjects without it (57.1% or 54.2%, respectively; proportion test, p=0.85) (Table 3). Only eight subjects who failed at the deep squat test had a personal history of knee surgery. Moreover, six subjects succeeded at the test in spite of having a history of knee surgery. Ankle sprain incidence was the same in subjects who failed or succeeded at the deep squat test (mean=2±2.4 or 2±3.2, respectively; Mann-Whitney test, p=0.36).

## Discussion

### Posture

The findings exhibited a very typical posture pattern – the depression of the scapula and shoulder of the preferred upper extremity combined with the elevation of the contra-lateral iliac crest (Table 1). Clinical findings were clearly evident and easily palpable through a close-fitting shirt, but structural scoliosis (accentuated asymmetry at the Adams test) was found in two subjects only. In all other cases remarkable functional scoliosis was classified. From our clinical experience these asymmetries are common in the general population, nevertheless they are more evident in our group of elite female volleyball athletes. The pattern of the elevated iliac crest on the left and a depressed shoulder girdle on the right could be linked to functional S-shaped scoliosis (or a scoliosis-like faulty posture) with the thoracic curve convex to the left and the lumbar curve convex to the right. Contrary to our findings, Kendall et al. (1993) described the prevalence of right thoracic-left lumbar functional scoliosis in right-handed individuals, but this pattern is more typical in subjects with structural idiopathic scoliosis. The reason for markedly bad posture in athletes could be an inadequate training load, an imbalance in their fitness training, imperfect technique and the high value of reaction forces as Langer and Langerova (2008) presumed in their work on elite high jumpers. Our findings of the presence of a depressed shoulder on the preferred side and/or functional scoliosis correspond to the findings of other authors. Kugler et al. (1996) observed the presence of a depressed “playing” shoulder and lateralised scapula in thirty competitive volleyball attackers (with a mean age of 25). These differences were of more significance in volleyball attackers with shoulder pain than in volleyball players without shoulder pain. In contrast to recreational athletes without any overhead sports activity, there were no significant differences in the comparison of the two shoulders. Henne (1998) described an extremely curved spine, slouching and protruding shoulders combined with weak shoulder blade fixation, muscle imbalance and changes in the passive locomotor system in volleyball athletes. Yoo et al. (2001) observed a higher incidence of truncal asymmetry and a scoliotic spinal column in one hundred and sixteen volleyball athletes. Sixty athletes (51.7%) exhibited more than 5 degrees of angle of trunk rotation measured by a scoliometer at the Adams test. Six athletes (5.17%) had an angle of 10–15 degrees, nobody had a higher angle. Yoo concluded that asymmetrical muscle development can produce mild scoliosis, however this doesn’t have the potential for severe progression as observed in some cases of idiopathic scoliosis. Wang and Cochrane (2001) found scapular asymmetry in five of sixteen athletes of England’s National Men’s Volleyball Squad using Kibler’s scapula lateral slide test. Moreover, they found a significantly restricted active range of shoulder internal rotation in the dominant arm. However, they did not find any significant association between mobility impairment and scapular asymmetry, and shoulder injury or pain. In another study, Wang et al. (2004) examined twelve boys and twelve girls of the Taiwanese National Junior Volleyball Teams. They did not find any obvious asymmetry in their upper trunk nor extremities by visual inspection. However, they found a significant decrease in the range of active shoulder internal rotation in the boys’ dominant upper extremity as compared to the non-dominant limb, but did not find this in the girls. Burkhart et al. (2003) described a typical SICK scapula syndrome (**S**capular malposition, **I**nferior medial border prominence, **C**oracoid pain and malposition, dys**K**inesis of scapular movement) on the dominant upper limb in “overhead” athletes, including volleyball players. Voralek et al., (2007) reported “scoliosis” in 43% of elite female volleyball players aged 15–19 years (with a mean of 16.3 years), but they did not mention the differentiation between real scoliosis and a “scoliosis like” posture. Oyama et al. (2008) reported more internally rotated and anteriorly tilted dominant-side scapula in “overhead” athletes including fifteen volleyball players. Grabara and Hadzik (2009) found more symmetrical positions of the shoulders and pelvis and more asymmetrical shoulder blades and waist triangles (I did not find the term “waist triangles” what does this mean?) in adolescent volleyball female athletes (13–16 years) than in their untrained peers. Kenanidis et al. (2008) did not observe the effect of sports activities on the degree of the main scoliotic curve.

Although observation alone is sufficient to diagnose scapular asymmetry (McFarland et al., 2001), the functional asymmetries between the preferred and non-preferred (dominant and non-dominant, respectively) upper extremity could be more precisely examined by means of isokinetic dynamometry. Wang and Cochrane (2001) found significantly stronger shoulder internal rotators in both the concentric and eccentric test and weaker external rotators in the concentric test in the dominant upper extremity. Their results are quite similar to those of other authors, e.g. Markou and Vagenas (2006) who found significantly higher magnitudes of concentric peak torque and adjacent dynamic parameters of the shoulder internal rotation and lower peak torque ratio of external/internal rotators in the dominant upper limb in twenty four elite male offensive players from Greek national volleyball division.

### Mobility

The forward bending test is widely used in clinical practice. Severe restriction (fingers above the navicular bone or lower) can happen due to several reasons, for example the presence of back or hip troubles, abdominal obesity, short upper extremities versus long lower extremities, mostly in children, soleus muscular tightness, etc. Nevertheless hamstring tightness is generally the most common. Our subjects exhibited hypermobility in the forward bending test significantly. These results could be expected due to age, sex, and high-level sport activity together with regular stretching. Short legs could be the potential cause of false hypermobility in the forward bending test. But we can exclude short legs in elite female volleyball athletes at first sight without calculating the proportional index of trunk versus legs.

On the other hand, the results of the deep squat test were surprising. More than half of the subjects failed the test and the prevalence of restriction was, paradoxically, significantly higher in subjects with hypermobility in the forward bending test. The most probable explanation for restriction in the deep squat test is soleus muscular tightness, because all of the other potential reasons such as obesity, balance disorders and knee or ankle lesions were excluded. Elite volleyball players could hardly have some severe balance disorders and their BMI 21.8±1.78 is far from representing obesity. Only 23.5% of the persons who failed in the deep squat test had a history of knee surgery; significantly more of them did not have any severe knee injury. Mean ankle sprain was the same in both the groups of the subjects who succeeded or failed at the deep squat test. So the tightness of the soleus muscle, having suffered from chronic overloading and/or a bad stretching method or system remained as the most probable cause. Moreover, stretching is regularly performed before each practice session and/or match, but the after-load relaxation exercises are often left out, probably due to tiredness and a lack of time.

Soleus muscular tightness (or shortness) is restricted not only to the deep squat test, but the forward bending test too. However, hypermobility in the forward bending test significantly prevailed in subjects who failed at the deep squat test, their ability to bend forward is mentioned above which means that there was some (over) compensation in the hamstrings, hip joint or spine in the forward bending test. It could be taken as physiological compensation for the soleus muscular shortness seen at first glance, but it could also be the origin of serious troubles in the lower back and other parts of the movement system. Moreover, it could be advanced by an inappropriate stretching system based on incorrect presumptions.

Contrary to our results Voralek et al. (2007) observed hamstring tightness in 90% (sic!), soleus muscular tightness in 7% and gastrocnemius muscular tightness in 90% (sic!) in elite female volleyball athletes aged 15–19 years (mean 16.3 years). Although the forward bending test used by us does not strictly target hamstrings, the significantly prevailing hypermobility in our group contradicts hamstring tightness, despite the potential compensation in the lumbar spine. Moreover, Voralek et al. (2007) did not describe the test. They only referred to Janda, whose description of the triceps surae muscular test was a little bit ambiguous and allowed various interpretations. Nevertheless findings of extremely high incidence of tight gastrocnemius contrary to low incidence of tight soleus muscles (see above) are very uncommon. In fact, DiGiovanni et al. (2002) proved the existence of isolated gastrocnemius contracture. We did not carry out discriminative tests for gastrocnemius and/or soleus muscular tightness in our group. However, our multiyear clinical experience and consultation with other experts reflects a higher incidence of soleus muscular tightness than in case of the gastrocnemius. Our observations are indirectly supported by histological findings. Gollnick et al. (1974) reported 80% (64–100%) of slow twitch fibres (Type I) in the human soleus muscle in contrast to 57% (34–82%) in the gastrocnemius muscle. Edgerton et al. (1975) observed 70% of slow twitch fibres in the soleus muscle, but only 50% in the gastrocnemius muscle. Similarly, Secher et al. (1982) reported a higher proportion of slow twitch fibres in the soleus muscle (71±5%) than in the gastrocnemius muscle (54±3.1%). A higher proportion of slow twitch fibres is related to muscle tightness according to Janda (Bänsch, 2005).

The discrepancy between hypermobility in the forward bending test and restriction in the deep squat test could have two main reasons. The first one is the chronic overload of the soleus muscle because of a multitude of jumps. The second one could be wrong stretching methods. Human psychological nature is to focus itself on rewarding activity and it could be more rewarding to repeat successful hamstrings stretching than the unsuccessful, uncomfortable and maybe painful stretching of the calf muscles.

## Conclusions

Our findings exhibited a “typical” postural pattern in right-handed elite female volleyball athletes with a higher acromion process, scapula and iliac crest on the left side than on the right side. This pattern was observed in fifty subjects (80.6%), significantly more frequently than other patterns. All but two cases could be classified as remarkable functional scoliosis without accentuated asymmetry by the Adams test.

A significant prevalence of hypermobility in the forward bending test was not surprising because of the common body composition in elite female volleyball athletes and regular stretching exercises. On the other hand, surprisingly more than half of the subjects did not succeed at the deep squat test. These poor results were not linked to age, history of knee surgery nor ankle sprain. Moreover, the hypermobility in the forward bending test significantly prevailed in those subjects who did not succeed at the deep squat test.

In our opinion, the cause of poor results at the deep squat test in elite female volleyball athletes is the tightness of the soleus muscle having suffered from chronic overloading and/or a bad stretching system. Inappropriate and/or insufficient compensatory exercise and an insufficient stretching system can cause marked postural asymmetry as well. A detailed examination of postural and muscular imbalance provided by an experienced physician or physiotherapist as well as individually tailored compensatory exercises and an effective stretching system can be strongly recommended to all athletes, not only to volleyball players.

## Figures and Tables

**Table 1:** Postural asymmetry and spiking arm

**Frontal plane asymmetry**					**Spiking arm**		
	
**Acromion**	**Scapula**	**Iliac crest**	*Sum*		**Right**	**Left**	*Proportion test significance*
higher on the							
		
left	left	left	50	80.6%	49	1	<0.0001
left	left	right	3		3	0	
neutral	neutral	left	2		2	0	
right	right	left	2		2	0	
right	right	right	2	19.4%	2	0	
neutral	neutral	neutral	1		1	0	
left	neutral	left	1		1	0	
right	right	neutral	1		0	1	
	
*Sum*			62		60	2	<0.0001
*Proportion test significance*				<0.0001			

**Table 2:** Mobility in the forward bending test and deep squat test

**Forward bending test**		**Deep squat test**		*Sum*
		**optimum**	**restriction**	
**general norm**	fingers to navicular bone	2	3	5
	fingers to floor	8	8	16
		35.7%	32.4%	33.9%
**hypermobility**	metatarsal heads to floor	4	1	5
	palms to floor	14	22	36
		64.3%	67.6%	66.1%
	*Sum*	28	34	62
	*Proportion test significance*	0.03	0.004	0.0003

**Table 3:** The effects of knee surgery on deep squat test results

**Deep squat test**	**healthy knee**		**knee surgery**		*Proportion test significance*
optimum	22	78.6%	6	21.4%	<0.0001
	45.8%		42.9%		0.85
restriction	26	76.5%	8	23.5%	<0.0001
	54.2%		57.1%		0.85

*Proportion test significance*	0.41	0.84	0.45	0.84	
